# Fever with pancytopenia: unusual presentation of extrapulmonary tuberculosis: a case report

**DOI:** 10.1186/s13256-018-1596-0

**Published:** 2018-03-06

**Authors:** Chamara Dalugama, Indika Bandara Gawarammana

**Affiliations:** Department of Medicine, University of Peradeniya, Peradeniya, Sri Lanka

**Keywords:** Extrapulmonary tuberculosis, Pancytopenia, Bone marrow, Granuloma

## Abstract

**Background:**

Tuberculosis is a major health problem in the developing world. Diagnosis of extrapulmonary tuberculosis is delayed because the presentation is nonspecific. Extrapulmonary tuberculosis can present with various hematological manifestations, including pancytopenia. Pancytopenia could be due to hypersplenism, maturation arrest, hemophagocytic lymphohistiocytosis, or infiltration of the bone marrow by caseating or noncaseating granulomas causing reversible or irreversible fibrosis.

**Case presentation:**

We report a case of a 56-year-old Sri Lankan Sinhalese man who presented with pyrexia of known origin with significant loss of weight and loss of appetite. He had mild pallor with mild hepatosplenomegaly. He had high inflammatory markers with pancytopenia in a peripheral blood smear. His chest radiograph was unremarkable, and he had a negative Mantoux test result. A diagnosis of disseminated tuberculosis was made on the basis of caseating tuberculous granulomas in the bone marrow.

**Conclusions:**

Disseminated tuberculosis remains a diagnostic challenge because the presentation is vague and nonspecific. In case of pyrexia of unknown origin with peripheral cytopenia, the possibility of disseminated tuberculosis should be considered, particularly in endemic areas. Simultaneous culture and histopathological examination of the bone marrow is important in such instances, because results of common tests such as chest radiography or Mantoux tests can be negative.

## Background

Tuberculosis is a major health problem in the developing world. Although pulmonary tuberculosis is promptly diagnosed and treated, diagnosis of extrapulmonary tuberculosis is often delayed because of its nonspecific presentation [1]. Extrapulmonary tuberculosis can present with various hematological manifestations, including pancytopenia [2, 3]. Pancytopenia can be due to hypersplenism [4], maturation arrest [4], hemophagocytic lymphohistiocytosis (HLH) [5, 6], or infiltration of the bone marrow by caseating or noncaseating granulomas causing reversible or irreversible fibrosis [7–12].

Owing to protean manifestations of disseminated tuberculosis, diagnosis remains a challenge to the treating physician. Patients presenting with pyrexia of known origin with peripheral cytopenia in an endemic region must alert the physician to the possibility of disseminated tuberculosis, and investigations including bone marrow need to be carried out promptly because delays in diagnosis carry high morbidity and mortality. This case report highlights that a high degree of clinical suspicion, followed by prompt diagnosis and treatment, resulted in an excellent outcome in a patient with disseminated tuberculosis.

## Case presentation

We report a case of a 56-year-old Sri Lankan Sinhalese man from the Kandy region who presented to the Peradeniya Teaching Hospital with a history of high-grade fever of 3 weeks’ duration associated with drenching night sweats. During this period, he had marked loss of appetite, and he had lost 6 kg of weight over the course of 3 weeks. He denied long-standing cough, alteration of bowel habits, or headache. His past medical history was unremarkable. He was a teacher by profession, a nonsmoker, and a social drinker. He denied any past history or contact history of tuberculosis.

On examination, he had mild pallor and was not icteric. He was febrile with a temperature of 39.8 °C. He had no lymphadenopathy. His cardiovascular and respiratory system examinations were unremarkable; his blood pressure was 100/60 mmHg. His abdominal examination revealed a soft, nontender abdomen with mild hepatosplenomegaly, soft in consistency. He did not have features of meningism. The results of his neurological examination, including higher functions, were unremarkable. His optic fundus was unremarkable.

A complete blood count revealed anemia with hemoglobin of 7.6 g/dl (reference range 14.0–17.5), leukopenia 840/μl (reference range 4000–10,000) with neutropenia of 640/μl, and a low platelet count of 112 × 10^3^/μl (reference range 150–450). Pancytopenia and reactive lymphocytes with profound neutropenia and moderate thrombocytopenia were evident in the patient’s blood workup. His C-reactive protein level was 206 mg/L with an erythrocyte sedimentation rate of 135 mm in the first hour. His blood culture and urine cultures were sterile. His chest radiograph was unremarkable. His renal function was normal. He had a low serum albumin level (26 g/dl) with a normal prothrombin time. His liver enzymes were elevated (alanine aminotransferase 318 U/L, aspartate aminotransferase 444 U/L), and his serum bilirubin level was normal. His lactate dehydrogenase concentration was 1620 U/L. Cerebrospinal fluid (CSF) analysis showed normal protein and sugar levels with five lymphocytes, and the patient’s CSF culture was sterile. The patient’s melioidosis serology was negative. The result of his Mantoux test was negative. An ultrasound scan showed mild hepatosplenomegaly with no intra-abdominal lymphadenopathy or solid organ lesions. His 2D echocardiogram was unremarkable. The results of contrast-enhanced computed tomography (CT) of the patient’s chest, abdomen, and pelvis were negative. His hepatitis profile, malaria film, and human immunodeficiency virus serology were negative.

The patient was started on intravenous meropenem 1 g every 8 hours empirically after blood and urine were taken for cultures. Intravenous teicoplanin was subsequently added to the treatment regimen. He continued to spike high fevers, and his condition deteriorated.

**Fig. 1:**
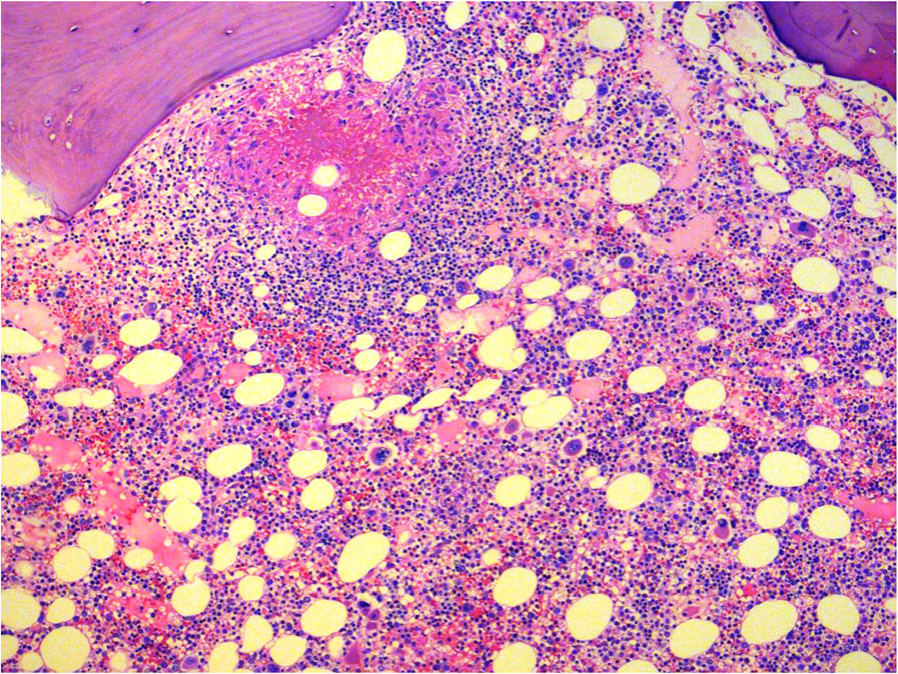
Histological preparation of the bone marrow showing diffuse infiltrate of large atypical cells withslightly nuclear pleomorphism and focal necrosis with caseating granulomas

A histological examination of a bone marrow biopsy was performed (Fig. 1). The pathology results showed a diffuse infiltrate of large atypical cells with slightly nuclear pleomorphism and focal necrosis with caseating granulomas. Results of acid-fast staining and polymerase chain reaction assays for *Mycobacterium tuberculosis* were both positive. The patient’s bone marrow adenosine deaminase level was 85 U/L. His lipid profile did not show high triglyceride levels, and his serum ferritin level was only mildly elevated (900 ng/ml).

The patient was started on standard antituberculosis treatment, including isoniazid, rifampicin, pyrazinamide, and ethambutol with intravenous steroids. Soon after starting the antituberculosis treatment, the patient complained of headache, and we found a tender area over the second lumbar vertebra. Magnetic resonance imaging of brain and pan-spinal imaging were arranged immediately, and they did not show evidence of tuberculoma or infective discitis. We could not find a primary lesion that led to disseminated tuberculosis, because results of a chest x-ray and contrast-enhanced CT of the chest, abdomen, and pelvis were also negative. The patient made a rapid clinical improvement, and he was fever-free after 3 days of treatment. His blood counts improved over the course of 1 week, and his liver enzyme levels normalized. A diagnosis of disseminated tuberculosis was made, and he was referred to a local chest clinic for continuation of antituberculosis drug treatment. He was seen in follow-up 3 months later in the medical clinic. He was continuing to receive antituberculosis drugs. He was well and free of fever and constitutional symptoms, with a weight gain of 5 kg over this period. His complete blood count showed a hemoglobin level of 12 g/dl, white blood cell count of 7000/μl, and a platelet count of 380 × 10^3^/μl. His antituberculosis treatment continued for 9 months. At the end of 9 months of antituberculosis treatment, he was back to his premorbid status with a hemoglobin level of 13.3 g/dl, and he was carrying out his normal activities.

## Discussion

Tuberculosis is a major public health problem in Sri Lanka. The incidence of active pulmonary tuberculosis has declined as a result of prompt diagnosis and treatment, but the incidence of extrapulmonary tuberculosis remains constant because the diagnosis is delayed owing to very nonspecific presentations [1]. We report a case of a patient with pyrexia of unknown origin who presented with peripheral cytopenia and high inflammatory markers. He had negative chest radiograph and negative Mantoux test results. Histopathology of his bone marrow demonstrated tuberculous granulomas with caseation. Our patient made a dramatic recovery with antituberculosis treatment. This case report is unique in that it describes a rare but recognized presentation of disseminated tuberculosis with pancytopenia and a dramatic response to the treatment following prompt diagnosis.

Extrapulmonary tuberculosis can present with various hematological manifestations. Singh *et al.* described various hematological manifestations in patients with both pulmonary and extrapulmonary tuberculosis [2, 3]. Normocytic normochromic anemia was the most common abnormality noted in all groups. Other hematological abnormalities of the white blood cells include leukopenia, neutropenia, lymphocytopenia, monocytopenia, leukocytosis, neutrophilia, lymphocytosis, and monocytosis. Pancytopenia was observed only in patients with disseminated/miliary tuberculosis. Platelet abnormalities were also very common. Thrombocytopenia was more common in patients with disseminated/miliary tuberculosis, whereas thrombocytosis was more common in patients with pulmonary tuberculosis.

Our patient had pancytopenia in a peripheral blood smear. Pancytopenia in disseminated tuberculosis is multifactorial. It can be due to hypersplenism, which was unlikely in our patient because his splenomegaly was mild and would not account for such a severe degree of pancytopenia [4]. Maturation arrest due to disseminated tuberculosis is rarely known to cause pancytopenia [4].

HLH should be considered in the differential diagnosis of patients with tuberculosis who present with cytopenia, organomegaly, and coagulopathy [5, 6]. The diagnosis was made in all previously reported cases on the basis of a constellation of fever, organomegaly, cytopenia(s), elevated serum ferritin and triglyceride levels with or without lower plasma fibrinogen, and demonstration of histiocytic hemophagocytosis on bone marrow examination [7]. The differential diagnosis of HLH was considered in the case of our patient, but his serum ferritin was mildly elevated with normal triglyceride levels and no bone marrow evidence of histiocytic hemophagocytosis.

Infiltration of the bone marrow by caseating or noncaseating granulomas causing reversible or irreversible fibrosis is another possible mechanism of pancytopenia in tuberculosis [7]. Our patient had diffuse infiltrate of large atypical cells with slightly nuclear pleomorphism and focal necrosis with caseating granulomas in the bone marrow. The other patients reported in the literature with disseminated/miliary tuberculosis with granulomas in the bone marrow had severe anemia, peripheral monocytopenia, and bone marrow histiomonocytosis, in contrast to the group of patients with disseminated/miliary tuberculosis without granulomas [2].

Tuberculosis is one of the most frequent causes of bone marrow granulomas; it constitutes 6–48% of the cases [8–10]. In cases of miliary tuberculosis, mostly the bone marrow biopsy will show granulomas (33–100%); caseation is uncommon (29%), and the presence of acid-fast bacilli detected by Ziehl-Neelsen staining is rare [11].

## Conclusions

Disseminated tuberculosis remains a diagnostic challenge because the presentations are nonspecific. Our patient had fever with cytopenia, a normal chest radiograph, and a negative Mantoux test result. Delay in diagnosis owing to lack of specific clinical features is the main reason for the poor prognosis in disseminated tuberculosis. Wang *et al.* described that simultaneous culture and histopathological examination of bone marrow in diagnosing disseminated tuberculosis is more sensitive than just performing a mycobacterial blood culture [12]. The favorable outcome of our patient was probably due to prompt diagnosis with early initiation of antituberculosis treatment.
